# Intersectional inequalities in advanced stage diagnosis of colorectal cancer in England: a cross-sectional study of National Cancer Registry data from 2013 to 2019

**DOI:** 10.1136/jech-2025-223740

**Published:** 2025-12-03

**Authors:** Claire Welsh, Andrew Bell, Natalie C Bennett

**Affiliations:** 1 National Disease Registration Service, NHS England, Leeds, UK; 2 Sheffield Methods Institute, The University of Sheffield, Sheffield, UK; 3 The University of Manchester, Manchester, UK

**Keywords:** Health inequalities, PUBLIC HEALTH, SCREENING

## Abstract

**Background:**

Inequalities in colorectal cancer (CRC) staging and outcomes exist across numerous sociodemographic axes. Early-stage CRC diagnosis is important for treatment success and survival. In this study, we investigate inequalities in CRC staging using registry data for 186 713 first-time CRC cancer diagnoses from 2013 to 2019 in England.

**Methods:**

We employ the novel Multilevel Analysis of Individual Heterogeneity and Discriminatory Accuracy (MAIHDA) approach to National Cancer Registry data. We investigate inequalities in CRC staging (early vs advanced stage) via a logistic MAIHDA. We examine a range of intersectional inequalities in CRC staging, across different age, ethnicity, gender and area-level deprivation groups.

**Results:**

Just over half of the staged cancers in the sample were diagnosed at advanced stage (62%). Results demonstrate notable inequalities in the risk of advanced CRC staging, with a gap of 17 percentage points between the strata with the lowest and highest predicted probability of advanced stage CRC diagnosis. These inequalities exist between age groups, ethnicity and deprivation level, with no evidence of gender-related inequalities when other variables are controlled. However, unexpectedly, we find these inequalities to be almost entirely additive in nature.

**Conclusions:**

These results suggest substantial inequalities in advanced stage CRC diagnosis exist, but that these are driven largely by universal processes of inequality, rather than disadvantages associated with single intersectional strata beyond an additive layering of disadvantage. Policy tools to encourage prompt screening engagement and symptom awareness campaigns in pre-screening age groups may benefit from considering the groups most disadvantaged by that additive layering.

**WHAT IS ALREADY KNOWN ON THIS TOPIC**

- Inequalities in colorectal cancer (CRC) staging exist across several important sociodemographic variables. However, these inequalities are typically examined individually, and their possible additive and multiplicative combinations remain largely unknown.

**WHAT THIS STUDY ADDS**

- Applying Multilevel Analysis of Individual Heterogeneity and Discriminatory Accuracy to the study of cancer stage at diagnosis, we find inequalities by age, ethnicity and area deprivation. However, the analysis reveals an almost entirely additive picture of inequalities in advanced stage CRC diagnosis, with minimal multiplicative effects.

**HOW THIS STUDY MIGHT AFFECT RESEARCH, PRACTICE OR POLICY**

- Policies and interventions aimed at improving symptom and risk factor awareness, particularly in those outside screening age, may benefit from considering the groups most disadvantaged by the additive combination of characteristics we identify.

## Introduction

 Colorectal cancer (CRC) is diagnosed in around 35 000 cases annually in England and is the fourth most common cancer in the UK.^1^ Large inequalities also exist in the uptake of testing, diagnosis, treatment and outcomes of CRC patients in England, across multiple spectra including socioeconomic deprivation, ethnicity and age.^2^,^5^ Males account for more than half of cases^6^ with incidence rates higher in white versus other ethnic groups,^2^ and higher with advancing age and in the most deprived places.^1^ Males are thought to have higher rates due to a combination of biology and lifestyle factors (diet, exercise, smoking and alcohol).^6^ Evidence also suggests that black patients and patients living in the most deprived areas are more likely to present with later stage CRCs than white patients from less deprived places^7^ and that survival is worse for patients living in more deprived areas.^8^

Cancers diagnosed later in their disease course often carry worse prognoses, due to limitations in treatment options and reductions in treatment efficacy. Diagnosis at an early stage is therefore associated with much better treatment outcomes and better survival rates.^9^ The UK’s cancer outcomes lag behind comparative countries’, which has been ascribed largely to the effects of later diagnosis.^9^ In addition, the burden of poor outcomes due to advanced diagnosis tends to fall more heavily on already deprived communities.^10^ Due to this, reducing advanced stage diagnosis is a key element of the NHS’s Long Term Plan.^11^ The stage of the cancer at diagnosis is a key point at which inequalities can emerge or persist. Advanced stage diagnosis of CRC could suggest poor access to primary healthcare, discrimination within primary or secondary care, limited access to national screening programmes, reduced health literacy, a lack of awareness of symptoms or reluctance to engage with health professionals more generally.^12 13^ These factors are likely to in part explain some of the inequalities observed in CRC and related outcomes. For example, difficulties accessing healthcare due to inflexible work could contribute to socioeconomic inequalities,^14^ while differential treatment recommendations,^15^ language barriers and culturally insensitive information campaigns and health systems could partially explain ethnic inequalities.^16^

While many studies have considered inequalities in cancer-specific outcomes in turn, few have applied an intersectional lens, considering how complex processes of privilege and disadvantage combine to produce cumulative and exacerbated health disadvantages for individuals at the intersection of multiple processes of disadvantage.^17 18^

Since December 2006, the NHS Bowel Cancer Screening Programme has been inviting people in England aged 60–69 to undergo screening once every 2 years, to improve diagnosis in asymptomatic people, increasing the proportion of patients diagnosed at early stages of disease. In 2021, eligibility was extended to those aged 50–74 in a phased rollout that completed in 2025.^19^ Uptake of free CRC screening has been unequal since it was first offered, including lower uptake in more deprived and ethnically diverse areas.^20^ In addition, awareness campaigns aimed at improving symptom knowledge and recognition in those aged 50+ are thought to have been broadly successful at improving symptom awareness.^21^ However, this may not necessarily have translated into greater CRC detection.^22^

Much is known about existing inequalities that are faced by CRC patients in England. However, an intersectional perspective may reveal previously unidentified heterogeneity in CRC inequalities. Interventions addressing single-dimension inequalities risk further disadvantaging cohorts of patients who experience multiple intersecting processes of disadvantage, often resulting in disproportionately worse outcomes. It is crucial that a more holistic understanding of the distribution of disadvantage is generated, such that future interventions are primed to succeed in reducing health inequalities.

This paper uses the innovative Multilevel Analysis of Individual Heterogeneity and Discriminatory Accuracy (MAIHDA)^23^ approach to assess the contribution of intersectionality to inequalities in CRC staging among patients in England. The method allows us to distinguish between additive inequalities (universal inequalities associated with sociodemographic variables, which layer together for specific combinations of those variables) and multiplicative inequalities (whereby the disadvantage faced by a group at a specific intersection is uniquely disadvantaged over and above what we would otherwise expect from only an additive combination of these characteristics). MAIHDA outperforms other quantitative methods often used for investigating intersectional inequalities^24^ and has been applied to a range of outcomes.

In this paper, we apply an intersectional lens to cancer stage at diagnosis, using an innovative, robust method to understand which complex combinations of identities are most at risk—a crucial question for health policymakers looking to reduce cancer incidence and improve survival. This method is descriptive, rather than causal, but allows the first investigations into the distribution of intersectional inequalities in CRC. This study aims to apply MAIHDA to interrogate the novel area of intersectional inequalities within cancer research and assesses the following research questions:

(RQ1) To what extent are intersectional groupings different from one another in their risk of advanced stage cancer diagnosis?

(RQ2) To what extent are there further multiplicative effects and if so, are specific strata especially advantaged or disadvantaged?

## Methods

### Data

England’s National Cancer Registration and Analysis Service (NCRAS) is part of the National Disease Registration Service, NHS England.^25^ Diagnoses of all registered colorectal tumours (based on the International Classification of Diseases V.10 codes C18, C19 and C20) between 2013 and 2019 were extracted from England’s registry data in December 2024, where the CRC diagnosis was each patient’s first malignant cancer diagnosis. The date range was chosen to maximise data availability while avoiding the effects of COVID on case ascertainment and subsequent changes to age range eligibility for screening.^19^ Diagnoses were only included if the registration was finalised, the patient’s age was recorded between 0 and 200 years (as per the NCRAS Counting Cases Standard Operating Procedure^26^), and complete information was available on ethnicity and gender. We include a flowchart detailing these sample restrictions in online supplemental appendix figure A1. Date of, and stage at diagnosis details were aligned with the Index of Multiple Deprivation score (IMD) for the patient’s Lower Super Output Area (LSOA) (average population 1000–3000 people^27^), based on their home postcode at diagnosis.

The variables describing the intersectional strata were decided in part based on the variables that previous published literature suggests are likely to be important, and what was available in the data. The variables are:

- Gender (male and female).
- Ethnicity (collapsed into white, black, Asian, other (including mixed)). Further details are provided in online supplemental appendix figure.
- Age group (0–49, 50–59, 60–69, 70–79, 80+).
- IMD (quintiles, where quintile 1 indicates the most deprived 20% of LSOAs in England).

These variables combine to produce 200 potential intersectional strata. In some cases (notably, age), inequalities in these variables may represent biological differences as well as differences in whether those groups are covered by the existing screening programmes. In other cases, these may represent social differences in an individual’s attitudes to healthcare, inequalities in access to care or discrimination against particular groups. Though coarse, our choice of these cut points was driven by balancing theory behind CRC staging inequalities and data availability. We expand on this further in the discussion and acknowledge that there may be significant within-strata social inequalities.

The outcome variable used is stage at diagnosis. This was dichotomised to early (stage 1 or 2) versus advanced (stage 3, 4 or unstaged), meaning our model considers inequalities in the odds of being diagnosed in the later stages compared with the earlier stages. We include unstaged cancers in the ‘advanced’ category due to the likelihood of diagnosis without an assigned stage being due to advanced disease. NCRAS published data showed a 5-year survival rate of unstaged patients to lie between the rates for stage 3 and 4 patients, further justifying this grouping.^28^ We include sensitivity analyses excluding unstaged cancers from our analysis (online supplemental tables A1–A3) and including unstaged cancers within the early stages 1 and 2 category (online supplemental tables A4–A6) in the appendix. The results from these were consistent with those presented below.

### Modelling

The MAIHDA approach takes a simple two-level model, where individuals are nested within their intersectional strata defined above. Since our outcome is binary, we use a logit link function. Two models are created. Model 1 is a ‘null’ two-level model, with no variables in the fixed part of the model other than the intercept.


$$logit\left[P\left(late_{ij}\right)\right]=\beta_{0}+u_{j}$$

here, ${late}_{ij}$ is a binary indicator of whether an individual *i* in intersectional stratum *j* was diagnosed early (0) or advanced (1). $\beta_{0}$ represents the log odds of the mean probability of advanced staging across all strata, while $u_{j}$ represents the differential from that mean for stratum *j*. We estimate the variance of $u_{j}$, assuming it is normally distributed with a mean of zero:


$$u_{j}∼N\left(0,\sigma_{u}^{2}\right)$$

In traditional MAIHDA, we would calculate the variance partitioning coefficient (VPC), which in a logit model (where the level 1 variance can be approximated to be that of a standard logistic distribution) is:


$$VPC=\frac{\sigma_{u}^{2}}{\sigma_{u}^{2}+3.29}$$

where 3.29 is the variance of a standard logistic distribution. Alternatively, for logit models, we could also calculate the median odds ratio (MOR)^29^ (see online supplemental appendix).

Model 2 is as model 1, but with the addition of the strata defining variables to investigate additive and multiplicative effects:


$$logit\left[P\left(late_{ij}\right)\right]=\beta_{0*}+\sum_{1}^{k}\beta_{k}X_{k}+u_{j*}$$

where $X_{k}$ represents k dummy variables for all the categories of gender, ethnicity, age and IMD (bar a reference category for each variable), each with a coefficient $\beta_{k}$ estimated. These coefficients provide the main, additive effects of these variables, as they would in a standard regression model. $u_{j}*$ now represents the remaining strata multiplicative effects once the additive effects in the fixed part of the model have been accounted for. As in model 1, these are assumed to be normally distributed and their variance estimated as:


$$u_{j*}∼N\left(0,\sigma_{u*}^{2}\right)$$

It is worth noting that we do not include any further control variables in our analysis. While the mechanisms by which that (dis)advantage may occur will likely include pathways involving other variables, including those variables in the model would control out those effects. This would not be desirable given our interest in describing, rather than explaining inequalities in CRC stage in this analysis.

It may be of interest to consider the extent to which (universal) additive effects can explain inequalities between strata, and the extent to which these inequalities are produced through strata-specific, multiplicative processes. For this, we calculate the proportional change in variance (PCV)—that is, the proportion of strata variance that is explained by additive effects.


$$PCV=\frac{\sigma_{u}^{2}-\sigma_{u*}^{2}}{\sigma_{u}^{2}}$$

Here, a low PCV would indicate substantial multiplicative effects, whereas a high PCV would suggest predominantly additive effects driving the strata inequalities. This will allow us to answer RQ2: we can consider the extent to which inequalities between strata are multiplicative (via the PCV), while also considering which specific strata have multiplicative effects (via estimates of $u_{j*}$).

Logistic models are fitted using a Laplace Approximation of maximum likelihood estimation using the R function ‘glmmTMB’.^30^

## Results

Data on 186 713 first-time CRC diagnoses (between 2013 and 2019) with complete case information were available for use in this study (from a total initial sample size of 365,900—see online supplemental appendix figure A1 for details). Over the 7-year data period, of the staged cancers recorded, just over half were diagnosed at an advanced stage (n=1 15 101, 61.7%). The distributions of key demographic variables are shown in table 1 (and online supplemental table A7 for the unfiltered dataset). As might be expected, of those in younger age groups included in the data who were not covered by the screening programme during the time of data collection, advanced stage diagnosis is more common than stage 1 and 2 (eg, for the 0 to 49 group, stage 1 and 2 n=4267 (31.3%) and advanced stage n=9359 (68.7%)). Furthermore, advanced stage diagnosis is slightly more common in people residing within the most and second most deprived LSOAs compared with earlier diagnosis (stages 1 and 2) (eg, most deprived stage 1 and 2 n=11752 (36.3%), advanced stage n=20662 (63.7%)). Ethnicity and gender distributions appear broadly similar across the two categories of stage at diagnosis.

**Table 1:** Characteristic descriptions across early (stages 1 and 2) versus advanced (stages 3, 4 and unstaged) stage colorectal cancer diagnosis in England from 2013 to 2019

	Stage 1, 2	Stage 3, 4 and unstaged	Overall
	(N=71 612)	(N=1 15 101)	(N=186 713)
5-year age band (years)			
0–49	4267 (31.3%)	9359 (68.7%)	13 626
50–59	8457 (34.6%)	15 966 (65.4%)	24 423
60–69	19 201 (41.7%)	26 800 (58.3%)	46 001
70–79	22 961 (42.0%)	31 728 (58.0%)	54 689
80+	16 726 (34.9%)	31 248 (65.1%)	47 974
Gender			
Female	31 789 (47.8%)	52 328 (62.2%)	84 117
Male	39 823 (38.8%)	62 773 (61.2%)	102 596
Ethnic group			
Asian	1840 (37.7%)	3036 (62.3%)	4876
Black	1031 (32.7%)	2123 (67.3%)	3154
Other	1041 (33.9%)	2026 (66.1%)	3067
White	67 700 (38.6%)	107 916 (61.4%)	175 616
Deprivation level			
1—Most deprived	11 752 (36.3%)	20 662 (63.7%)	32 414
2	12 958 (37.1%)	21 931 (62.9%)	34 889
3	15 084 (39.0%)	23 631 (61.0%)	38 715
4	15 887 (39.3%)	24 545 (60.7%)	40 432
5—Least deprived	15 931 (39.6%)	24 332 (60.4%)	40 263

The minimum number of cases per stratum (n=200) was 8 (range 8 to 6739, mean=933.57). Just one stratum contained fewer than 10 cases. The count and proportion of strata containing different sample sizes are shown in table 2. The observed (grand) stratum mean was 64.8% advanced diagnoses, with an SD of 7.49 (distribution is shown in online supplemental figure A2). Parameter estimates for both model 1 and model 2 are shown in table 3.

**Table 2:** Summary of counts per stratum

Sample size per stratum	Number of strata	% of strata
100 or more	88	44
50 or more	161	80.5
30 or more	184	92
20 or more	192	96
10 or more	199	99.5
Less than 10	1	0.5

**Table 3:** MAIHDA model 1 (null) and 2 (additive) results.

Predictors	Model 1				Model 2			
	OR	Standard error	95% CI	P value	OR	Standard error	95% CI	P value
(Intercept)	1.79	0.03	1.72 to 1.86	**<0.001**	1.33	0.03	1.27 to 1.39	**<0.001**
White	*Reference*				*Reference*			
Asian					0.98	0.03	0.92 to 1.04	0.535
Black					1.19	0.05	1.10 to 1.28	**<0.001**
Other					1.15	0.05	1.07 to 1.25	**<0.001**
70–79	*Reference*				*Reference*			
0–49					1.55	0.04	1.47 to 1.63	**<0.001**
50–59					1.35	0.03	1.29 to 1.41	**<0.001**
60–69					1.00	0.02	0.96 to 1.04	0.901
80+					1.35	0.03	1.29 to 1.41	**<0.001**
Female	*Reference*				*Reference*			
Male					0.99	0.01	0.96 to 1.02	0.545
5—least deprived	*Reference*				*Reference*			
1—most deprived					1.13	0.03	1.08 to 1.18	**<0.001**
2					1.10	0.03	1.05 to 1.15	**<0.001**
3					1.03	0.02	0.98 to 1.07	0.255
4					1.01	0.02	0.96 to 1.05	0.722
**Random effects**								
L2 variance	0.04				0.00002			
VPC	0.01				0.00			
Strata N	200				200			
Observations	186 713				186 713			

Bold p-values indicate significance at p<0.05 level.

MAIHDA, Multilevel Analysis of Individual Heterogeneity and Discriminatory Accuracy; VPC, variance partitioning coefficient.

**Figure 1:**
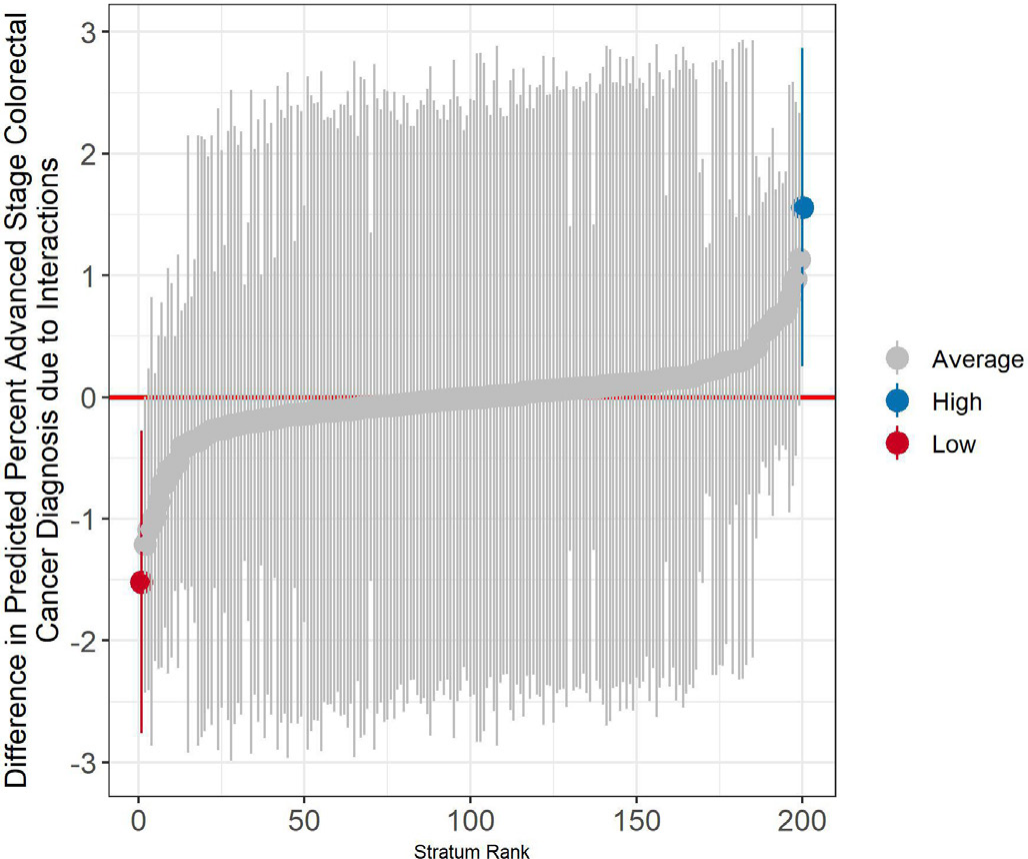
Caterpillar plot showing the stratum-specific residuals from model 2 of the predicted proportion diagnosed with colorectal cancer at an advanced stage (stage 3, 4 and unstaged) in England from 2013 to 2019. Strata (n=200) are comprised of the intersections of age, gender, ethnicity and the Index of Multiple Deprivation.

In our null model (model 1) some stratum-level variance is identified (0.04), indicating that there are some differences in CRC staging between strata, addressing RQ1. Calculating the MOR (see Larsen and Merlo^29^ and online supplemental appendix) results in an OR value of 1.26. In other words, if we took two randomly selected strata, on average, the less advantaged strata would have 26% greater odds of advanced-stage diagnosis compared with the more advantaged strata. Online supplemental figure A3 provides a visualisation of the predicted proportion of advanced stage diagnoses by strata, showing a difference of 17.08 percentage points (on the dependent variable’s probability scale) between the stratum with the lowest versus the highest predicted probability. To answer RQ2, inclusion of the covariates comprising the strata in model 2 results in a reduction of the stratum-level variance to almost zero. This suggests that the variance in cancer staging at the stratum level is almost entirely explained by additive inequalities and that there are no significant multiplicative effects. Table 3 shows the results for each of these axes comprising the strata. These results suggest that ethnicity, more deprived versus less deprived areas and most categories of age significantly predict advanced stage CRC diagnosis, and this is reflected in the highest and lowest strata predictions, shown in table 4. However, we find that gender does not significantly predict advanced stage diagnosis. Furthermore, we find just two strata with statistically significant interaction effects (see figure 1): the white, 80+, female, highest IMD stratum was the largest positive residual, while the white, 80+ male, lowest IMD was the largest negative residual. A positive residual indicates greater likelihood of advanced stage diagnosis than would have been expected based on the additive effects. However, it is worth noting that, although MAIHDA to some extent corrects for multiple testing, this correction will not be complete in the presence of significant two-way interactions in the data.^31^ As such, these results should not be overinterpreted.

**Table 4:** Six highest and lowest ranked predicted stratum means of the probability of advanced stage diagnosis (stage 3 or 4) of colorectal cancer in England between 2013 and 2019 (whereby strata are defined by intersections of age group, gender, ethnicity and IMD quintile)

Rank	Gender	Ethnic group	IMD* quintile (1=most, 5=least)	Age group	Count	Predicted proportion of advanced stage diagnoses	95% CI	
1	Male	Asian	5	60–69	95	56.1	53.55	58.65
2	Male	Asian	4	60–69	129	56.14	53.61	58.68
3	Male	White	5	70–79	6552	56.29	55.23	57.35
4	Female	Asian	5	70–79	67	56.39	53.81	58.96
5	Male	Asian	4	70–79	106	56.48	53.96	59.01
6	Male	Asian	5	70–79	77	56.56	54.00	59.13
200	Female	Black	1	0–49	100	73.18	70.90	75.46
199	Male	Black	1	0–49	98	73.02	70.74	75.31
198	Female	Black	2	0–49	91	72.83	70.54	75.12
197	Female	Other	1	0–49	80	72.8	70.49	75.11
196	Male	Black	2	0–49	74	72.71	70.40	75.01
195	Male	Other	1	0–49	87	72.45	74.78	74.78

IMD, Index of Multiple Deprivation.

Results of our analysis excluding unstaged cancers (online supplemental tables A1–A3) are broadly similar in terms of estimate size and direction, and in terms of very small interactive effects, though we find no statistically significant residuals in this model.

## Discussion

In this study, we employ a large and unique dataset, pooling English cancer registry data from 2013 to 2019 to investigate potential intersectional inequalities in CRC staging across four axes of inequality: gender, ethnicity, age and IMD. These axes are commonly proposed to be associated with inequalities in cancer screening participation.^2 6 8^ We employ the novel MAIHDA approach to examine the intersection of these inequalities. MAIHDA allows us to robustly uncover inequalities and interactions between complex subgroups produced through high-order interactions and test for the presence of multiplicative effects and is the first study to use MAIHDA in the field of cancer epidemiology and to consider these interactive effects, offering a rich and nuanced picture of cancer staging inequalities.

We find substantial inequalities in advanced stage CRC diagnosis across the intersectional groups included in our analysis, with a difference of 17 percentage points between the strata with the lowest and highest predicted probability. Our study reveals inequalities broadly in line with those acknowledged in the existing literature. We find reduced odds of being diagnosed with advanced stage CRC for all other quintiles of area deprivation compared with the most deprived (except quintile 2). This is in line with other studies indicating lower CRC screening participation in more deprived areas^32^ and higher CRC mortality.^33^ This finding may also be a result of factors such as barriers to healthcare, including difficulties accessing care around inflexible or insecure work,^14^ which could delay help-seeking.

In addition, we also find significant inequalities by ethnicity, with black and other groups having higher odds of advanced stage CRC diagnosis, though CIs around both estimates are wide. This is especially the case for the ‘other’ group, likely reflecting the heterogeneity of ethnicities grouped under this heading. Our findings are broadly in line with wider evidence, which suggests that minority ethnic groups are more likely to have lower screening uptake^34^ and that specific ethnic groups may be less aware of symptoms and screening availability.^35^ While we were unable to examine potential explanations for these inequalities in our analysis, it is likely that they have been in part produced through barriers such as language, health literacy and cultural norms,^16 36^ as well as structural processes including discrimination and prejudice, which may influence screening uptake or screening recommendation^15 37^ trust in healthcare providers, and individual behaviours including diet and exercise.

Furthermore, we find that the odds of advanced staged CRC diagnosis are greater in age groups not covered by routine screening or targeted by recent symptom awareness campaigns. The odds are especially high for the 0 to 49 age group. This may reflect that CRC has become more common in younger age groups in recent years.^10^ Finally, we find no evidence of inequalities by gender when the other explanatory variables are included in the model. While evidence suggests higher incidence of CRC in men, staging appears to be broadly similar in men and women.^6^

Notably, we find very little evidence of multiplicative inequalities. It should be noted that, in MAIHDA analyses, it is unusual to find such a small VPC model 2. Just two strata had statistically significant residuals. This suggests that the inequalities we observe in CRC staging are largely due to an additive accumulation of disadvantage.

It is important to highlight that the lack of multiplicative effects identified in this study does not prove the absence of intersectional inequalities in CRC staging. Our findings show that disadvantage does accumulate, with groups disadvantaged by multiple factors (ie, minority ethnic groups in more deprived areas at ages outside of routine screening coverage) most at risk of advanced diagnosis. It is also possible that interactive associations exist between important variables we have not been able to include in our analysis. For example, we were not able to assess social support, which is known to be relevant for screening participation^38^ and, therefore, likely to be an indicator of advanced stage diagnosis.

There are several important caveats and limitations to acknowledge. First, we have employed MAIHDA descriptively here, and we make no claims about possible processes by which some groups are more likely to be diagnosed at an advanced stage. Rather, MAIHDA provides a unique tool for describing inequalities and should form part of evidence triangulation.

In addition, there is significant within-group variability, which we have not examined and, relatedly, the grouping of ethnic groups into broader categories is problematic. For example, in our Asian category, we include Indian, Pakistani, Bangladeshi and Chinese ethnicities. These ethnic groups are all different in terms of language, culture and origin and are likely to have different probabilities of advanced CRC stage as evidence suggests that inequalities in screening uptake exist across these groups.^39^ Our choice of the coarseness of the categories of the variables comprising our strata is a careful balance of data availability and sample size. Investigation of other inequalities identifiable via further or differing categorisation is an important avenue for future research. Furthermore, we use data pooled from 2013 to 2019 on CRC cancer staging. We chose 2019 as a cut-off to exclude possible COVID-19 pandemic-related impacts as well as recent changes to the age range for eligibility.^19^ However, it is likely that these inequalities have shifted over this 7-year period and that the inequalities we observe may not reflect current inequalities. Drivers of changes may include altered demographics (eg, an ageing population), increased public awareness through high-profile diagnoses in the media or further targeted publicity campaigns, inequity in postpandemic health service recovery and other unpredictable factors.

Evidence suggests that between 70% and 75% of CRC cases are thought to be associated with modifiable risk factors (such as diet, exercise, smoking and alcohol consumption), compared with 25%–30% attributable to non-modifiable and genetic factors.^40^ We did not examine modifiable factors in our analysis as they were not available to us in the data. It is likely that these risk factors are unevenly socially patterned, and understanding this could help to explain some of the inequalities we observe. Future research should therefore seek to examine the contribution of modifiable risk factors to these intersectional inequalities.

Our data went as far as 2019. However, it would be interesting to look at whether the patterns that we have uncovered here remain consistent during and after the COVID-19 pandemic, or whether the pandemic has introduced additional inequalities/disadvantages for particular groups. At present, there is not enough post-COVID data published by NDRS to answer this question, and future work to consider this question when that data are available would be highly valuable.

Finally, income data were unavailable in our dataset. We use LSOA level IMD as a proxy (common practice in health inequalities studies). While IMD tells us important information about socioeconomic inequalities, which would otherwise not be possible to assess, ecological measures of socioeconomic deprivation likely capture different processes of disadvantage to that of individual socioeconomic status measures. Furthermore, other processes of disadvantage are also likely to be at play, which we were not able to capture in our analysis, such as discrimination, including related to language or faith.

## Conclusion

Using a large sample of nationwide registry data, we identify noteworthy inequalities in CRC staging across age, area deprivation and ethnicity. The results highlight the multidimensional nature of disadvantage and the need to consider this comprehensively when understanding inequalities in advanced stage CRC diagnoses. We find these inequalities are mostly additive in nature.

Readers might be tempted to interpret this to mean that policies can be broadly targeted based on single factors, and the results certainly suggest that individual factors would help to identify where the need for policy intervention is greatest across the population. In this case, a focus of policy on younger groups, the two most deprived IMD quintiles and black/other ethnicities might well be worthwhile. However, we would urge caution for a few reasons. First, our analysis considers the rate of advanced stage diagnosis *out of those who are diagnosed* and not the rates of occurrence *in the general population*. For instance, our non-significant sex coefficient does not negate past findings that CRC is more common in men in the general population. Second, often the effects of policies do not necessarily match the inequalities that they aim to address. Further work could be done considering intersectional patterning of the *effects* of policies and information campaigns (eg, see Jolidon *et al*^41^ for work in progress in this area). Furthermore, policies and interventions aiming to improve prompt screening engagement, symptom and risk factor awareness, particularly in the groups mentioned above, may benefit from considering the groups most disadvantaged by the additive layering of characteristics we identify.

## Supplementary material

10.1136/jech-2025-223740 online supplemental file 1

## Data Availability

Data may be obtained from a third party and are not publicly available.
