# Supplementary material for: Intersectional inequalities in advanced stage diagnosis of colorectal cancer in England: a cross-sectional study of National Cancer Registry data from 2013 to 2019
Source: J Epidemiol Community Health. 2025 Dec 3;80(3):e223740. doi: 10.1136/jech-2025-223740 (PMC13018759; doi:10.1136/jech-2025-223740)
Supplement: online supplemental file 1 [file jech-80-3-s001.pdf]

Appendix

Additional methods detail

Ethnicity coding

Ethnicity was available to us in the categories provided by the Census [1]. We therefore code our aggregated ethnicity categories as follows:

White: English, Welsh, Scottish, Northern Irish or British, Irish, Gypsy or Irish Traveller, Roma, Any other White background

Black: Caribbean, African, Any other Black, Black British, or Caribbean background

Asian: Indian, Pakistani, Bangladeshi, Chinese, Any other Asian background

Other: Arab, Any other ethnic group, White and Black Caribbean, White and Black African, White and Asian, Any other Mixed or multiple ethnic background

Ideally, we would have used more fine-grained ethnicity categorisations. However, we had to balance the sample size we had available to us with the questions and categories we were able to model. This is particularly an issue with logit models, where small group sizes can result in convergence issues as well as imprecise estimates. These convergence issues prevented us from conducting sensitivity analyses involving more fine grained ethnicity groupings.

Calculating the Median Odds Ratio (MOR)

The MOR can be interpreted as the median of the odds ratio between two randomly selected strata. It can be calculated as:

$$MOR = \exp[\sqrt{2 * 0.6745 * \sigma_u^2}]$$

Where 0.6745 is the 75th percentile of the cumulative distribution function of the Normal distribution N(0,1). As a comparison, the VPC is calculated as

$$VPC = \frac{\sigma_u^2}{\sigma_u^2 + 3.29}$$

Given both the MOR and VPC are functions only of σ_u^2 the MOR is a direct transformation of the VPC to a more interpretable scale; for instance, a VPC of 0.01 (or 1%) would be calculated from a strata-level variance of 0.04, and be equivalent to a MOR of 1.25.

Figure A1 – Sample flow chart

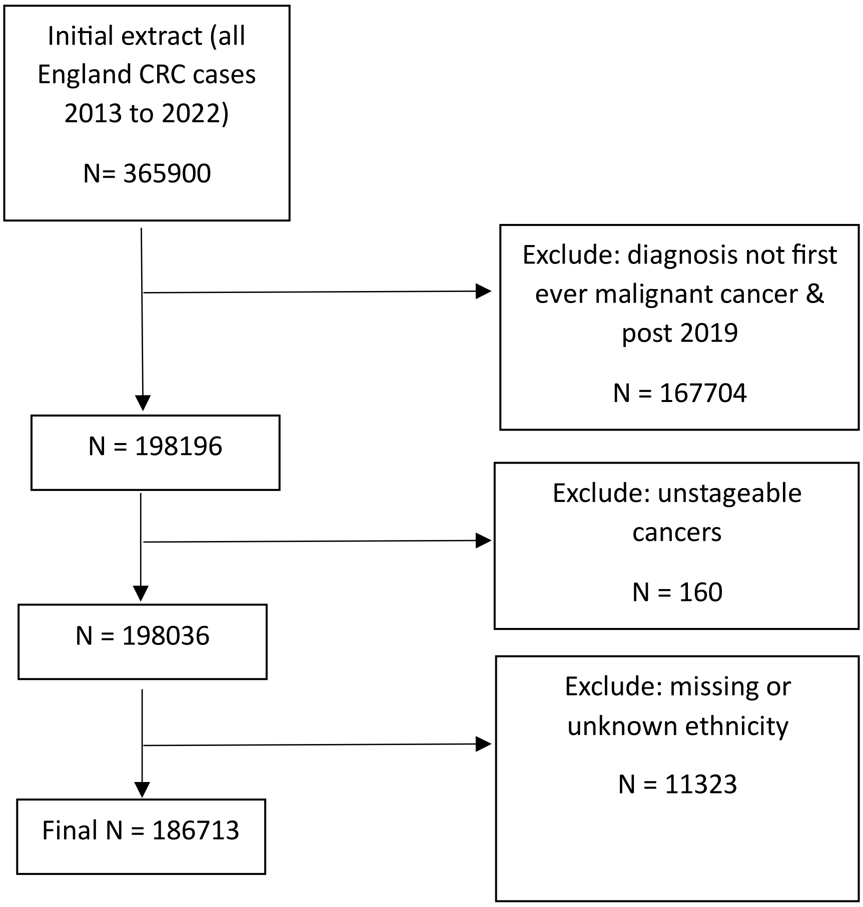

Figure A2 - Histogram of the mean percentage of late-stage (stage 3 or 4) colorectal cancer diagnoses in England between 2013 and 2019, for each of the 200 intersectional strata (composed of the intersections of age group, gender, ethnicity and IMD quintile)

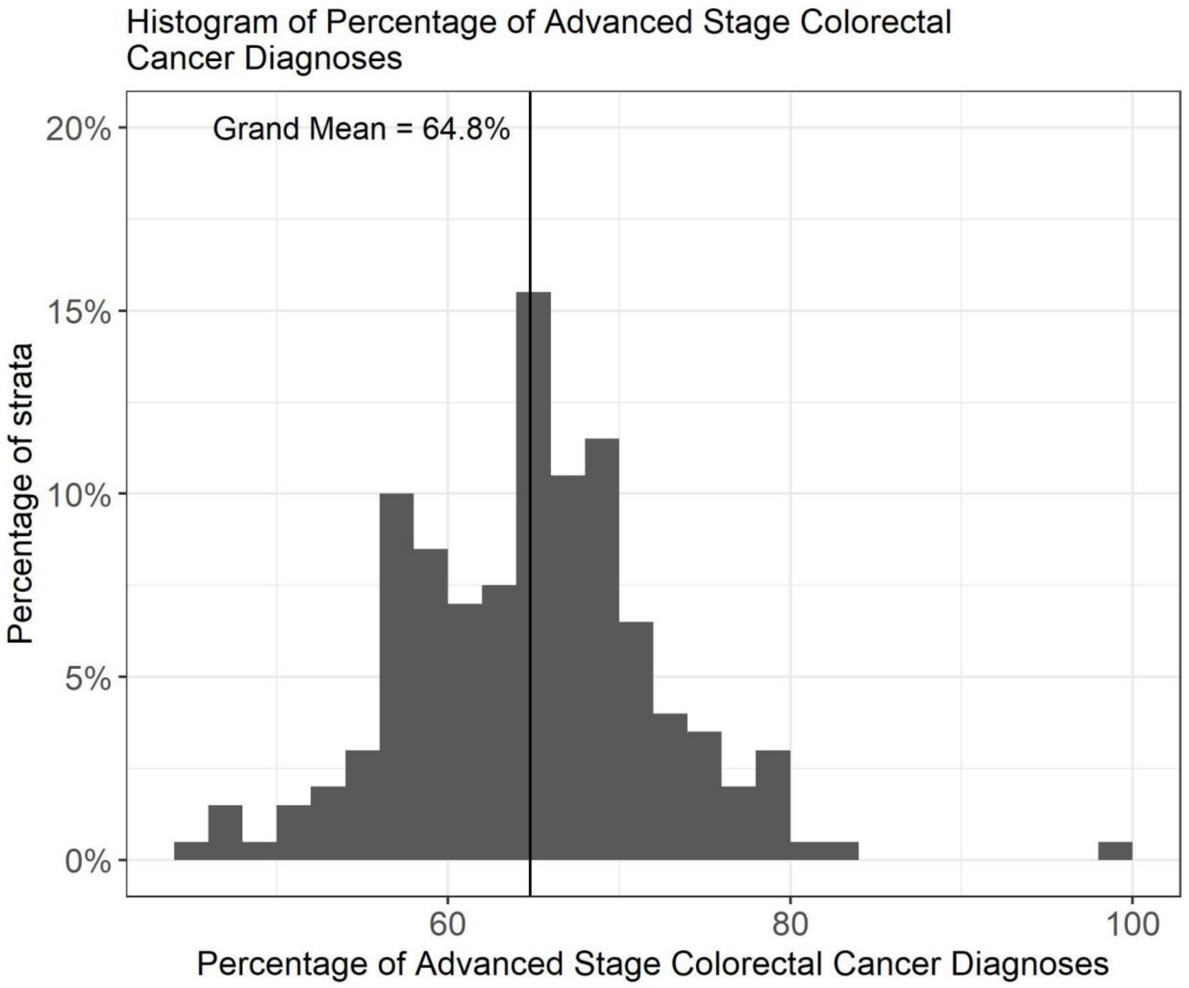

Figure A3 - Caterpillar plot showing stratum specific predicted proportion of advanced stage (stages 3 or 4) colorectal cancer diagnoses in England between 2013 and 2019, for each of the 200 intersectional strata (composed of the intersections of age group, gender, ethnicity and Index of Multiple Deprivation quintile)

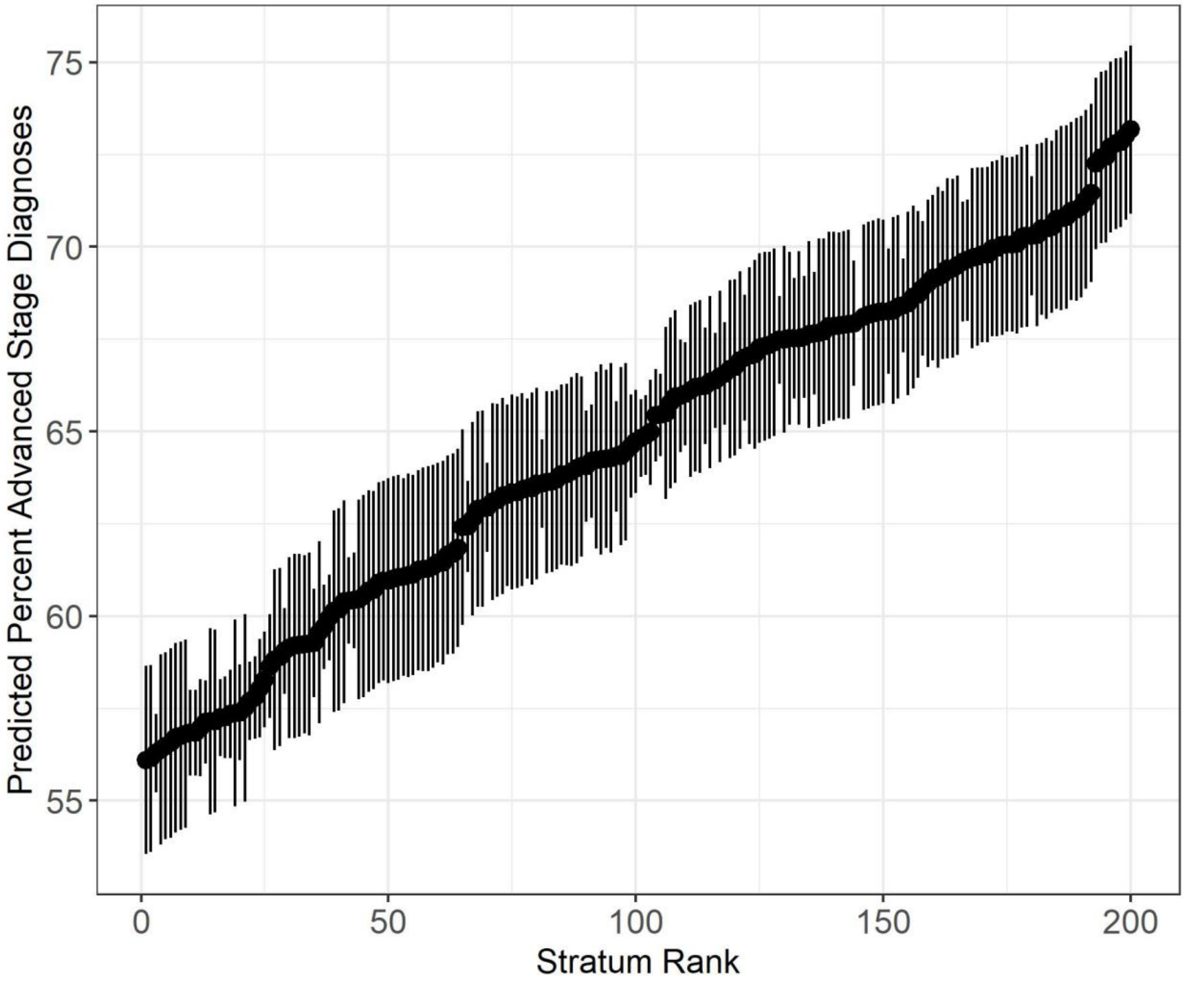

Supplementary Analysis 1

Excluding unstaged cancers – whereby ‘advanced’ stage cancers include only stages 3 and 4 (unstaged cancers are excluded from the analysis), and ‘early’ stage cancers include stages 1 and 2.

Table A1 - Characteristic descriptions across early (stages 1 and 2) versus advanced (stages 3 and 4) stage colorectal cancer diagnosis in England from 2013 to 2019

	**Stage 1, 2**	**Stage 3, 4**	**Overall**
	(N=29379)	(N=39341)	(N=68720)
**5-Year Age Band (years)**			
**0 to 49**	4267 (6.0%)	7972 (8.5%)	12239 (7.4%)
**50 to 59**	8457 (11.8%)	14242 (15.2%)	22699 (13.7%)
**60 to 69**	19201 (26.8%)	23652 (25.3%)	42853 (25.9%)
**70 to 79**	22961 (32.1%)	26922 (28.7%)	49883 (30.2%)
**80 plus**	16726 (23.4%)	20875 (22.3%)	37601 (22.8%)
**Gender**			
**Female**	31789 (44.4%)	41330 (44.1%)	73119 (44.2%)
**Male**	39823 (55.6%)	52333 (55.9%)	92156 (55.8%)
**Ethnic group**			
**Asian**	1840 (2.6%)	2452 (2.6%)	4292 (2.6%)
**Black**	1031 (1.4%)	1744 (1.9%)	2775 (1.7%)
**Other**	1041 (1.5%)	1655 (1.8%)	2696 (1.6%)
**White**	67700 (94.5%)	87812 (93.8%)	155512 (94.1%)
**Deprivation level**			
**1 - Most deprived**	11752 (16.4%)	16843 (18.0%)	28595 (17.3%)
**2**	12958 (18.1%)	17810 (19.0%)	30768 (18.6%)
**3**	15084 (21.1%)	19370 (20.7%)	34454 (20.8%)
**4**	15887 (22.2%)	19963 (21.3%)	35850 (21.7%)
**5 - Least deprived**	15931 (22.2%)	19677 (21.0%)	35608 (21.5%)

Table A2 - MAIHDA model 1 (null) and 2 (additive) results

	Model 1				Model 2			
Predictors	OR	Std. Error	95% CI	p	OR	Std. Error	95% CI	p
(Intercept)	1.46	0.03	1.40 – 1.52	**<0.001**	1.98	0.04	1.89 – 2.07	**<0.001**
White	*Reference*				*Reference*			
Asian					0.94	0.03	0.88 – 1.00	**0.048**
Black					1.17	0.05	1.08 – 1.27	**<0.001**
Other					1.12	0.05	1.04 – 1.21	**0.005**
70 to 79	*Reference*				*Reference*			
0 to 49					0.90	0.02	0.86 – 0.95	**<0.001**
50 to 59					0.66	0.01	0.64 – 0.69	**<0.001**
60 to 69					0.63	0.01	0.61 – 0.66	**<0.001**
80 plus					0.68	0.01	0.65 – 0.71	**<0.001**
Female	*Reference*				*Reference*			
Male					1.02	0.01	1.00 – 1.04	0.109
1 - most deprived	*Reference*				*Reference*			
2					0.97	0.02	0.93 – 1.00	**0.036**
3					0.91	0.01	0.88 – 0.94	**<0.001**
4					0.90	0.01	0.87 – 0.92	**<0.001**
5 – least deprived					0.88	0.01	0.85 – 0.91	**<0.001**
**Random effects**								
L2 variance	0.04				0.00			
VPC	0.01				0.00			
Strata N	200				200			
Obs	165275				165275			

Table A3 - Six highest and lowest ranked predicted stratum means of the probability of advanced stage diagnosis (stage 3 or 4) of colorectal cancer in England between 2013 and 2019 (whereby strata are defined by intersections of age group, gender, ethnicity and IMD quintile)

**Rank**	**Gender**	**Ethnic Group**	**IMD* Quintile** (5 = least, 1 = most)	**Age Group**	**count**	**Predicted proportion of advanced stage diagnoses**	**95% CI**	
1	female	Asian	5	70 to 79	58	50.93	49.25	52.61
2	male	Asian	5	70 to 79	68	51.34	49.66	53.01
3	female	Asian	4	70 to 79	66	51.35	49.67	53.03
4	male	Asian	4	70 to 79	95	51.75	50.09	53.42
5	female	Asian	3	70 to 79	86	51.79	50.12	53.47
6	female	Asian	5	60 to 69	58	52.08	50.40	53.77
200	male	Black	1	0 to 49	93	70.16	68.35	71.97
199	female	Black	1	0 to 49	89	69.82	68.00	71.64
198	male	Black	2	0 to 49	61	69.42	67.58	71.26
197	male	Other	1	0 to 49	82	69.25	67.40	71.10
196	female	Black	2	0 to 49	85	69.08	67.23	70.92
195	female	Other	1	0 to 49	69	68.91	67.04	70.77

***Index of Multiple Deprivation (IMD)**

Supplementary analysis 2

Including unstaged cancers in the early category – whereby 'advanced' stage cancers include only stages 3 and 4, and 'early' stage cancers include stages 1 and 2 and unstaged cancers.

Table A4 - Characteristic descriptions across early (stages 1, 2 and unstaged) versus advanced (stages 3 and 4) stage colorectal cancer diagnosis in England from 2013 to 2019

	**Stage 1, 2 + advanced**	**Stage 3, 4**	**Overall**
	(N=93,050)	(N=93,663)	(N=186,713)
**5-Year Age Band (years)**			
**0 to 49**	5654 (6.1%)	7972 (8.5%)	13626 (7.3%)
**50 to 59**	10181 (10.9%)	14242 (15.2%)	24423 (13.1%)
**60 to 69**	22349 (24.0%)	23652 (25.3%)	46001 (24.6%)
**70 to 79**	27767 (29.8%)	26922 (28.7%)	54689 (29.3%)
**80 plus**	27099 (29.1%)	20875 (22.3%)	47974 (25.7%)
**Gender**			
**Female**	42787 (46.0%)	41330 (44.1%)	84117 (45.1%)
**Male**	50263 (54.0%)	52333 (55.9%)	102596 (54.9%)
**Ethnic group**			
**Asian**	2424 (2.6%)	2452 (2.6%)	4876 (2.6%)
**Black**	1410 (1.5%)	1744 (1.9%)	3154 (1.7%)
**Other**	1412 (1.5%)	1655 (1.8%)	3067 (1.6%)
**White**	87804 (94.4%)	87812 (93.8%)	175616 (94.1%)
**Deprivation level**			
**1 - Most deprived**	15571 (16.7%)	16843 (18.0%)	32414 (17.4%)
**2**	17079 (18.4%)	17810 (19.0%)	34889 (18.7%)
**3**	19345 (20.8%)	19370 (20.7%)	38715 (20.7%)
**4**	20469 (22.0%)	19963 (21.3%)	40432 (21.7%)
**5 - Least deprived**	20586 (22.1%)	19677 (21.0%)	40263 (21.6%)

Table A5 - MAIHDA model 1 (null) and 2 (additive) results

	**Model 1**				**Model 2**			
Predictors	OR	Std. Error	95% CI	p	OR	Std. Error	95% CI	p
(Intercept)	1.10	0.02	1.06 – 1.15	<0.001	1.00	0.01	0.97 – 1.03	0.805
White	Reference				Reference			
Asian					0.91	0.03	0.86 – 0.96	0.001
Black					1.11	0.04	1.03 – 1.19	0.004
Other					1.05	0.04	0.98 – 1.13	0.189
70 to 79	Reference				Reference			
0 to 49					1.45	0.03	1.40 – 1.51	<0.001
50 to 59					1.44	0.02	1.39 – 1.48	<0.001
60 to 69					1.09	0.01	1.06 – 1.12	<0.001
80 plus					0.80	0.01	0.78 – 0.82	<0.001
Female	Reference				Reference			
Male					1.05	0.01	1.03 – 1.07	<0.001
1 - most deprived	Reference				Reference			
2					0.97	0.02	0.94 – 1.00	0.093
3					0.95	0.01	0.92 – 0.97	<0.001
4					0.92	0.01	0.90 – 0.95	<0.001
5 – least deprived					0.91	0.01	0.88 – 0.94	<0.001
**Random effects**								
L2 variance	0.05				0.00			
VPC	0.01				0.00			
Strata N	200				200			
Obs	186713				186713			

Table A6 - Six highest and lowest ranked predicted stratum means of the probability of advanced stage diagnosis (stage 3 or 4) of colorectal cancer in England between 2013 and 2019 (whereby strata are defined by intersections of age group, gender, ethnicity and IMD quintile)

**Rank**	**Gender**	**Ethnic Group**	**IMD* Quintile** (5 = least, 1 = most)	**Age Group**	**Count**	**Predicted proportion of advanced stage diagnoses**	**95% CI**	
1	female	Asian	5	80 plus	31	39.62	38.09	41.15
2	female	Asian	4	80 plus	50	40.03	38.50	41.56
3	female	Asian	3	80 plus	72	40.58	39.04	42.11
4	male	Asian	5	80 plus	58	40.78	39.23	42.32
5	male	Asian	4	80 plus	71	41.19	39.65	42.74
6	female	Asian	2	80 plus	79	41.28	39.74	42.83
200	male	Black	1	0 to 49	98	62.78	60.92	64.64
199	male	Black	1	50 to 59	134	62.52	60.73	64.32
198	male	Black	2	0 to 49	74	62.15	60.28	64.03
197	male	Black	2	50 to 59	122	61.91	60.09	63.72
196	female	Black	1	0 to 49	100	61.64	59.76	63.52
195	male	Black	3	0 to 49	38	61.47	59.56	63.38

***Index of Multiple Deprivation (IMD)**

Table A7 - Characteristic descriptions across early (stages 1 and 2) versus advanced (stages 3, 4 and unstaged) stage colorectal cancer diagnosis in England from 2013 to 2019, using an un filtered initial dataset.

	**Stage 1, 2**	**Stage 3, 4 & unstaged**	**Overall**
	(n=133,577)	(n=210,993)	(n=344,570)
**5-Year Age Band (years)**			
**0 to 49**	6437 (4.8%)	14674 (7.0%)	21111 (6.1%)
**50 to 59**	13346 (10.0%)	25517 (12.1%)	38863 (11.3%)
**60 to 69**	33074 (24.8%)	45155 (21.4%)	78229 (22.7%)
**70 to 79**	45628 (34.2%)	61044 (28.9%)	106672 (31.0%)
**80 plus**	35092 (26.3%)	64603 (30.6%)	99695 (28.9%)
**Gender**			
**Female**	58367 (43.7%)	94799 (44.9%)	153166 (44.5%)
**Male**	75210 (56.3%)	116194 (55.1%)	191404 (55.5%)
**Ethnic group**			
**Asian**	3002 (2.2%)	5254 (2.5%)	8256 (2.4%)
**Black**	1819 (1.4%)	3905 (1.9%)	5724 (1.7%)
**Other**	1885 (1.4%)	3649 (1.7%)	5534 (1.6%)
**White**	126871 (95.0%)	198185 (93.9%)	325056 (94.3%)
**Deprivation level**			
**1 - Most deprived**	20833 (15.6%)	36327 (17.2%)	57160 (16.6%)
**2**	23867 (17.9%)	39407 (18.7%)	63274 (18.4%)
**3**	28134 (21.1%)	43542 (20.6%)	71676 (20.8%)
**4**	29942 (22.4%)	45843 (21.7%)	75785 (22.0%)
**5 - Least deprived**	30801 (23.1%)	45874 (21.7%)	76675 (22.3%)

References

1 Gov.uk. Ethnicity facts and figures: List of ethnic groups. n.d.
